# Furmonertinib for EGFR-mutant advanced non-small cell lung cancer: a glittering diamond in the rough of EGFR-TKI

**DOI:** 10.3389/fphar.2024.1357913

**Published:** 2024-02-19

**Authors:** Jianghua Ding, Xingjing Ding, Jiao Zeng, Xiaoqun Liu

**Affiliations:** ^1^ Hematology and Oncology Department, Jiujiang University Affiliated Hospital, Jiujiang, Jiangxi, China; ^2^ Oncology Department, First Affiliated Hospital of Nanchang University, Nanchang, Jiangxi, China; ^3^ Department of Graduate, Gannan Medical University, Ganzhou, Jiangxi, China; ^4^ Respiratory Department, Jiujiang University Affiliated Hospital, Jiujiang, Jiangxi, China

**Keywords:** furmonertinib, epidermal growth factor receptor, TKI, advanced, non-small cell lung cancer

## Abstract

The third-generation EGFR-TKIs, such as osimertinib, aumolertinib, and furmonertinib, have been recommended as the preferred treatment for EGFR-mutant advanced non-small cell lung cancer (NSCLC). Among them, furmonertinib shows several advantages in terms of clinical efficacy. Firstly, compared to osimertinib and aumolertinib, furmonertinib was the first EGFR-TKI with median progression-free survival (mPFS) of over 20.0 m (20.8 m) for advanced NSCLC with classical EGFR-mutations. Furthermore, furmonertinib achieved a mPFS of 18.1 m in advanced NSCLC with unfavorable prognostic factors, such as the 21 L858R mutation and central nervous system (CNS) metastasis, which is unrivalled by osimertinib. Secondly, furmonertinib is the only FDA-approved EGFR-TKI for breakthrough therapy in newly-diagnosed advanced NSCLC with EGFR ex20ins mutation. Thirdly, the relatively longer mPFS of 20.8 m was observed in furmonertinib compared to osimertinib and aumolertinib (15.2 m and 15.3 m) in EGFR-mutant advanced NSCLC with CNS metastases. More importantly, the efficacy of furmonertinib increases within the dose range of 80–240 mg per day. Finally, furmonertinib can be an optional treatment for advanced NSCLC patients who develop resistance to osimertinib or aumolertinib. In conclusion, furmonertinib may be a glittering star in the field of EGFR-TKI, which requires further exploration and expansion.

## Introduction

The discovery of the first driver gene, the epidermal growth factor receptor (EGFR), has ushered in the era of personalized and precise medical treatment for non-small cell lung cancer (NSCLC). The prevalence of EGFR mutation is 10%–20% in Caucasian populations, but can be as high as 50% in Asian patients (Wu and Zhou, 2023). EGFR tyrosine kinase inhibitor (EGFR-TKI) agents have revolutionized the therapeutic strategy for NSCLC with EGFR mutations. Since the introduction of the first EGFR-TKI (gefitinib), there has been a continuous emergence of newer generations of EGFR-TKI over the past 2 decades, including the 1st-generation (gefitinib, erlotinib, icotinib), the 2nd-generation (afatinib, dacomitinib), and the 3rd-generation (osimertinib, aumolertinib, furmonertinib). According to the 2023 Chinese Society of Clinical Oncology (CSCO) guideline for NSCLC, the preferred upfront treatment is the 3rd-generation EGFR-TKI, as it has demonstrated excellent efficacy compared with the other generations of EGFR-TKI (Chinese Association for Clinical Oncologists, 2023). It is worth noting that furmonertinib appears to outshine the other two EGFR-TKIs (i.e., osimertinib and aumolertinib). In this section, we will explore the specific advantages of furmonertinib.

## Furmonertinib for classical EGFR-mutant advanced NSCLC

The EGFR exon 19 deletion (19 del) and exon 21 L858R mutation (21 L858R) account for approximately 90% of EGFR mutations, which are commonly referred to as classical EGFR mutations. A network meta-analysis found that furmonertinib, osimertinib, and aumolertinib may represent the optimal treatment for Asian patients, due to prolonged survival, greater tumor burden response, and lower risk of adverse events (Chen et al., 2023). For advanced NSCLC with classical EGFR-mutations, the first-line treatment of osimertinib, aumolertinib, and furmonertinib achieved median progression-free survival (mPFS) of 18.9, 19.3, and 20.8 months, respectively, in comparison with gefitinib or erlotinib (Soria et al., 2018; Shi et al., 2022a; Lu et al., 2022) (Table 1). At present, furmonertinib is the first agent passing the mark of 20.0 m PFS among available EGFR-TKIs for NSCLC patients (Shi et al., 2022b). For 19 del mutation subgroup, all three 3rd-generation EGFR-TKIs showed marked advantage of mPFS over the 1st-generation EGFR-TKI (gefitinib or erlotinib). In terms of PFS, furmonertinib decreased the risk of disease progression to 65%, marginally higher than that of osimertinib (57%) and aumolertinib (61%) (Ramalingam et al., 2020; Shi et al., 2022b; Lu et al., 2022).

**TABLE 1:** Prospective and retrospective clinical study of third-generation EGFR-TKI treatment for advanced EGFR-mutant NSCLC.

Author/Year	Study/Phase	Title	Sample number	Intervention/Treatment	mPFS	mOS
Soria et al. (2018)	FLAURA (Phase 3)	Osimertinib in Untreated EGFR-Mutated Advanced Non-Small-Cell Lung Cancer	279 Vs. 277	Osimertinib Vs. Gefitinib/Erlotinib	18.9 m Vs. 10.2 m (HR = 0.46, 0.37–0.57)	38.6 m Vs. 31.8 m (HR = 0.80, 0.64–1.00)
			19 del subgroup: 158 Vs. 155	Osimertinib Vs. Gefitinib/Erlotinib	HR = 0.43 (0.32–0.56)	HR = 0.68 (0.51–0.90)
			21 L858R subgroup: 97 Vs. 90	Osimertinib Vs. Gefitinib/Erlotinib	HR = 0.51 (0.36–0.71)	HR = 1.00 (0.71–1.40)
			CNS-M subgroup: 53 Vs. 63	Osimertinib Vs. Gefitinib/Erlotinib	15.2 m Vs. 9.6 m (HR = 0.47, 0.30–0.74)	-
Cheng et al. (2021)	FLAURA China (Phase3)	Osimertinib Versus Comparator EGFR TKI as First-Line Treatment for EGFR-Mutated Advanced NSCLC: FLAURA China, A Randomized Study	71 Vs. 65	Osimertinib Vs. Gefitinib/Erlotinib	17.8 m Vs. 9.8 m (HR = 0.56, 0.37–0.85)	33.1 m Vs. 25.7 m (HR = 0.85, 0.56–1.29)
			19 del subgroup: 36 Vs. 33	Osimertinib Vs. Gefitinib/Erlotinib	HR = 0.41 (0.22–0.77)	HR = 0.61 (0.32–1.18)
			21 L858R subgroup: 35 Vs. 32	Osimertinib Vs. Gefitinib/Erlotinib	HR = 0.69 (0.39–1.21)	HR = 1.02 (0.59–1.78)
Lu et al. (2022)	AENEAS (Phase 3)	AENEAS: A Randomized Phase III Trial of Aumolertinib Versus Gefitinib as First-Line Therapy for Locally Advanced or Metastatic Non-Small-Cell Lung Cancer With EGFR Exon 19 Deletion or L858R Mutations	214 Vs. 215	Aumolertinib Vs. Gefitinib	19.3 m Vs. 9.9 m (HR = 0.46, 0.36–0.60)	-
			19 del subgroup: 140 Vs. 141	Aumolertinib Vs. Gefitinib	20.8 m Vs. 12.3 m, (HR = 0.39, 0.28–0.54)	-
			21 L858R subgroup: 74 Vs. 74	Aumolertinib Vs. Gefitinib	13.4 m Vs. 8.3 m (HR = 0.60, 0.40–0.89)	-
			CNS-M subgroup: 56 Vs. 59	Aumolertinib Vs. Gefitinib	15.3 m Vs. 8.2 m (HR = 0.38, 0.24–0.60)	-
Shi et al. (2022a)	FURLONG (Phase 3)	Furmonertinib (AST2818) *versus* gefitinib as first-line therapy for Chinese patients with locally advanced or metastatic EGFR mutation-positive non-small-cell lung cancer (FURLONG): a multicentre, double-blind, randomised phase 3 study	178 Vs. 179	Furmoenrtinib Vs. Gefitinib	20.8 m Vs. 11.1 m (HR = 0.44, 0.34–0.58)	-
			19 del subgroup: 91 Vs. 92	Furmoenrtinib Vs. Gefitinib	HR = 0.35 (0.23–0.53)	-
			21 L858R subgroup: 87 Vs. 87	Furmoenrtinib Vs. Gefitinib	HR = 0.54 (0.37–0.77)	-
			21 L858R in CNS-M subgroup: 30 Vs. 30	Furmoenrtinib Vs. Gefitinib	18.1 m Vs. 8.2 m (HR = 0.39, 0.19–0.80)	-
			CNS-M subgroup: 65 Vs. 62	Furmoenrtinib Vs. Gefitinib	20.8 m Vs. 9.8 m (HR = 0.40, 0.23–0.71)	-
Yasuda et al. (2021)	UMIN000031929	A phase I/II study of osimertinib in EGFR exon 20 insertion mutation-positive non-small cell lung cancer	14	Osimertinib (80 mg/d)	3.8 m	15.8 m
Yang et al. (2021)	--	Osimertinib for Chinese advanced non-small cell lung cancer patients harboring diverse EGFR exon 20 insertion mutations	62	Osimertinib (80 or 160 mg/d)	2.3 m (80 mg: 1.8 m; 160 mg: 2.5 m, *p* = 0.161)	-
van Veggel et al. (2020)	-	Osimertinib treatment for patients with EGFR exon 20 mutation positive non-small cell lung cancer	21	Osimertinib (80 or 160 mg/d)	3.6 m	8.7 m
Deng et al. (2022)	-	Aumolertinib-based comprehensive treatment for an uncommon site of EGFR exon 20 insertion mutations with multiple metastases non-small cell lung cancer: a case report	1	Aumolertinib (110 mg/d)	10.0 m	-
Sa et al. (2023)	FAVOUR (Phase 1)	A real-world study of the efficacy and safety of furmonertinib for patients with non-small cell lung cancer with EGFR exon 20 insertion mutations	53	Furmonertinib (80, 160 or 240 mg/d)	6-months PFS: 69.4% (53.7%–85.1%)	-
Villaruz et al. (2023)	NCT03434418 (Phase 2)	A single-arm, multicenter, phase II trial of osimertinib in patients with epidermal growth factor receptor exon 18 G719X, exon 20 S768I, or exon 21 L861Q mutations	17	Osimeritnib (80 mg/d)	10.5 m	13.8 m
Zhao et al. (2023)	NCT03127449 (Phase 2a)	Central nervous system efficacy of furmonertinib (AST2818) in patients with EGFR T790M mutated non-small cell lung cancer: a pooled analysis from two phase 2 studies	52	Furmonertinib (40, 80, 160 or 240 mg/d)	CNS-PFS: (40mg, 2.8m; 80mg, 11.6m; 160mg, 19.3m; 240 mg: NR)	-
	NCT03452592 (Phase 2b)					
Hu et al. (2023)	-	Efficacy and safety of re-challenging 160 mg furmonertinib for advanced NSCLC after resistance to third-generation EGFR-TKIs targeted agents: A real-world study	39	Furmonertinib (160 mg/d)	4.7 m (maximum: 13.8 m)	-
			IP group: 22 EP group: 17	Furmonertinib (160 mg/d)	5.5 m Vs. 3.2 m (*p* = 0.0018)	9.8 m Vs. 6.7 m (*p* = 0.021)

Compared to subgroup of 19 del mutation, the 21 L858R mutation subgroup was often accompanied by concomitant mutations, such as TP53, PIK3CA, BRAF, MET, MYC, CDK6, and CTNNB1, indicating a worse prognosis (Li and Cui, 2020). The FLAURA study showed that osimertinib prolonged the mPFS (HR = 0.51, 0.36–0.71). However, it did not prolong the median overall survival (mOS) of the 21 L858R mutation subtype (HR = 1.00, 0.71–1.40) compared with gefitinib/erlotinib treatment (Ramalingam et al., 2020). The analysis of the Chinese group of the FLAURA study revealed that osimertinib did not exhibit a survival advantage in terms of PFS (HR = 0.69, 0.39–1.21) and OS (HR = 1.02, 0.59–1.78) over gefitinib or erlotinib in the 21 L858R subtype (Cheng et al., 2021). However, in the AENEAS and FURLONG studies, aumolertinib and furmonertinib significantly increased the PFS of 21 L858R subtype compared to gefitinib (HR = 0.60, 0.40–0.89; HR = 0.54, 0.37–0.77) (Shi et al., 2022b; Lu et al., 2022). In particular, longer PFS was observed in furmonertinib treatment than in gefitinib treatment (18.1 vs. 8.2m, *p* = 0.0076) in the subgroup of both 21 L858R mutation and central nervous system (CNS) metastasis in FURLONG study (Shi et al., 2022a) (Table 1). The PFS of 18.1 months is the longest survival in available studies for advanced EGFR-mutant NSCLC with unfavorable prognostic factors. Therefore, for advanced classical EGFR-mutant NSCLC, furmonertinib demonstrates a distinct advantage in terms of clinical efficacy, particularly for the 21 L858R mutation and CNS metastasis.

## Furmonertinib for advanced NSCLC harboring EGFR exon 20 insertions

As the third most common type of EGFR mutation, exon 20 insertions (EGFR ex20ins) represent up to 12% of all EGFR-mutant NSCLC with 5-year OS of 8%. Patients with NSCLC harboring ex20ins exhibit a mPFS of 3.4–6.9 months and an objective response rate (ORR) of 23%–29% when treated with platinum-based chemotherapy. In contrast, the use of immune checkpoint inhibitors as second-line treatment resulted in only an ORR of 10% and a mPFS of 2.7 months for these patients (Hou et al., 2022). Several clinical studies have demonstrated that osimertinib (80 mg/d) for EGFR ex20ins only achieved an ORR of 0%–6.5% and PFS of 2.3–3.8 months, indicating a very limited efficacy of osimertinib (van Veggel et al., 2020; Yang et al., 2021; Yasuda et al., 2021) (Table 1). Only a case report has been published on the use of aumolertinib for advanced NSCLC with EGFR ex20ins, demonstrating a PFS of 10 months (Deng et al., 2022). Encouragingly, a phase Ib study (FAVOUR) reported the efficacy of furmonertinib in 53 patients with advanced NSCLC harboring EGFR ex20ins. The ORR was 37.7%, the disease control rate (DCR) was 92.5%, and the 6-month PFS rate was 69.4% (95% CI 53.7%–85.1%). Moreover, the ORR increased with the dose intensity, with rates of 25.0% for 80 mg/d, 39.5% for 160 mg/d, and 42.9% for 240 mg/d (*p* = 0.816). Importantly, patients with CNS metastases possessed similar ORR to those without CNS metastases (33.3% vs. 40.6%, *p* = 0.773). The efficacy of furmonertinib was independent of the location of the EGFR ex20ins mutation (Sa et al., 2023) (Table 1). Thus, in October 2023, the U.S. Food and Drug Administration granted breakthrough therapy status to furmonertinib for the treatment of newly-diagnosed advanced NSCLC with EGFR ex20ins mutation.

In advanced NSCLC patients with EGFR ex20ins who progressed after chemotherapy, furmonertinib demonstrated an ORR of 37.7% (Sa et al., 2023). This was similar to the ORR for amivantamab (40%) and CLN-081 (41%), higher than that for mobocertinib (28%) and poziotinib (15%), but lower than that for sunvozertinib (59.8%). Amivantamab has both EGFR and MET-related toxicities, with 66% of infusion-related reactions. The dose reduction rates for mobocertinib and poziotinib were 72% and 25% due to adverse events, respectively. Additionally, 20.2% of patients treated with sunvozertinib required dosage reduction, and 7.9% required discontinuation (Park et al., 2021; Zhou et al., 2021; Elamin et al., 2022). In summary, furmonertinib, a promising 3rd-generation EGFR-TKI, has a wide therapeutic window (80 mg–240 mg) for advanced NSCLC patients with ex20ins. It also has a good safety profile with no dose-limiting toxicities.

## Furmonertinib for uncommon EGFR-mutant advanced NSCLC

In addition to the aforementioned mutations, there are also uncommon EGFR mutations, including exon 18 G719X, exon 20 S768I, and exon 21 L861Q mutations, which account for 10%–20% of all EGFR mutations in NSCLC. A *post hoc* analysis was conducted, which enrolled three trials of afatinib for 38 patients with NSCLC who had the three uncommon EGFR mutations. The ORR was 71% (95% CI: 54%–84%), and the mPFS was 10.7 months (95% CI: 5.6–14.7 months) (Yang et al., 2015). A single-arm, phase II study enrolled 17 patients with uncommon EGFR-mutations who were treated with osimertinib. The ORR was 47% [95%CI: 23%–72%], the mPFS was 10.5 months (95% CI 5.0–15.2 months), and the mOS was 13.8 months (95% CI 7.3–29.2 months) (Table 1) (Villaruz et al., 2023), indicating the limited efficacy of osimertinib against the type of NSCLC.

A preclinical data demonstrated furmonertinib targeting G719S (Ba/F3 cellular IC50 = 12.4 nM), S7681 (Ba/F3 cellular IC50 = 21.6 nM) and L861Q (Ba/F3 cellular IC50 = 3.8 nM) (Musib et al., 2022). Zhao Y *et al.* reported a female advanced NSCLC with original EGFR L861Q mutation and secondary MET amplification, who progressed after osimertinib plus chemotherapy, and afatinib treatment. The patient received combined treatment of furmonertinib with crizotinib. Encouragingly, she achieved partial remission with a PFS of 6 months, indicating the potential candidate to overcome the resistance of osimertinib and afatinib (Zhao et al., 2023). The results strongly suggested that furmonertinib could be regarded as a potent agent against the uncommon EGFR mutations. A clinical study (NCT05548348) is in recruiting stage in which furmonertinib (160 mg/day) is employed to treat advanced NSCLC patients harboring uncommon EGFR mutations (Table 2). The study is still ongoing, and the results are worth expecting.

**TABLE 2:** Main TRAEs of the 3rd-generation EGFR-TKI.

TRAEs types	Osimertinib (FLAURA-China)	Aumolertinib (AENEAS) (%)	Furmonertinib (FURLONG) (%)
Diarrhea	24%	16.4	27
prolonged Q-T interval	10%	10.7	10
Skin rash	37%	23.4	17
Leucopenia	41%	23.8	17
Thrombocytopenia	28%	22	11
Anemia	38%	20.1	17
ILD	3%	0.9	0.5
CPK elevation	-	35.5	6

Abbreviation: Vs., versus; CNS-M, central nervous system metastases; IP group, intracranial progression group; EP group, extracranial progression group; DFS, disease free survival; TRAEs, Treatment-related adverse events; ALT, alanine aminotransferase; AST, aspartate aminotransferase; ITD, interstitial lung disease; CPK, creatine phosphokinase.

## Furmonertinib for advanced NSCLC with central nervous system metastases

Central nervous system (CNS) is a common site of metastases in patients with EGFR-mutated NSCLC, and is associated with a poor prognosis. For all patients with CNS metastases, osimertinib showed a better mPFS compared to gefitinib/erlotinib (15.2 vs. 9.6 months, *p* < 0.001; HR = 0.47, 0.30–0.74) in FLAURA study (Soria et al., 2018). Similarly, the mPFS for the aumolertinib and gefitinib group was 15.3 months and 8.2 months, respectively (HR = 0.38, 0.24–0.60) (Lu et al., 2022). The FURLONG study showed that furmonertinib prolonged the mPFS to 20.8 months compared to gefitinib (9.8 months) for patients with CNS metastases (HR = 0.40, 0.23–0.71) (Shi et al., 2022b). Comparatively, furmonertinib exhibited superior efficacy in terms of PFS compared to osimertinib and aumolertinib. Furthermore, a *post hoc* analysis was conducted in 52 NSCLC patients with EGFR-mutation and CNS metastases who received furmonertinib treatment ranging from 40 mg/d to 240 mg/d. The CNS-ORR were 65% for 80 mg/d group and 85% for 160 mg/d group. The median CNS-PFS for 40 mg/day, 80 mg/d, 160 mg/day, and 240 mg/day was 2.8 months, 11.6 months, 19.3 months and not reached (NR) respectively, suggesting a dose-dependent effect of furmonertinib (Hu et al., 2023) (Table 1). These findings supported furmonertinib as a preferred treatment option for EGFR-mutant NSCLC with concurrent CNS metastases.

## Furmonertinib for advanced NSCLC after osimertinib/aumolertinib resistance

Osimertinib commonly exhibits an initial positive response in advanced NSCLC with classical EGFR mutation. However, acquired resistance to osimertinib is inevitable. Recently, researchers have investigated the therapeutic strategy of substitution among the third-generation EGFR-TKI. In areal-world study, 39 patients with EGFR-mutated NSCLC received the innovative re-challenge of furmonertinib (160 mg/d) after resistance to osimertinib or aumolertinib (Table 1) (Qi et al., 2023). The DCR was 79.5% and the mPFS was 4.7 months with the maximum PFS of nearly 13.8 months, which outperformed previously reported strategies. Encouragingly, the patients in the intracranial progression (IP group, N = 22) had markedly higher mPFS and mOS than those in the extracranial progression (EP group, N = 17) (5.5 vs. 3.2 months, *p* = 0.0018; 9.8 vs. 6.7 months, *p* = 0.021). Thus, furmonertinib displayed favorable efficacy in patients with IP, suggesting that furmonertinib possessed greater penetration of blood-brain barrier compared to osimertinib or aumolertinib.

The underlying mechanism may be associated with the unique pharmacological and physiological features of furmonertinib. As a novel third-generation EGFR-TKI, furmonertinib introduces the innovative trifluoroethoxypyridine structure. Firstly, *in vivo* studies have shown that furmonertinib irreversibly inhibits EGFR-sensitive and resistant mutations, such as G719X, 19 del, 21 L858R, L861Q, and T790M (Musib et al., 2022). In terms of pharmacology, the active metabolite AST5902 has similar anticancer activity to the furmonertinib prototype (Meng et al., 2022; Musib et al., 2022), indicating “dual antitumor activity”. Secondly, the introduction of the trifluoroethoxypyridine structure significantly improved the lipid solubility of the furmonertinib prototype and its metabolites. An *in vitro* study found that the intracranial uptake of furmonertinib and AST5902 was four times higher than that of osimertinib (Shi et al., 2020), suggesting “dual brain entry”. Thirdly, unlike osimertinib, furmonertinib and AST5902 are not substrates of the efflux transporter (i.e., P-gp) and thus easily cross the blood-brain barrier, supporting high selectivity for intracranial metastases (Shi et al., 2020). Finally, compared to EGFR mutants, furmonertinib and AST5902 have a lower inhibitory capability on wild-type EGFR and other related receptors (Meng et al., 2022; Musib et al., 2022), which partly explain the good safety profile of high-dose furmonertinib for human. These findings suggested that furmonertinib (160 mg/d) may be a novel rescue strategy for those patients after osimertinib or aumolertinib failure.

## Safety profile of furmonertinib

Besides clinical efficacy, the safety profile is another important issue to consider in clinical settings. As shown in (Table 2), the diarrhea incidence of furmonertinib was marginally higher than osimertinib and aumolertinib (27% vs. 24% vs. 16.4%). The incidences of Q-T intervals prolongation were nearly similar among the three drugs. More importantly, the lowest frequencies of other treatment-related adverse effects (TRAEs) were observed in the group of furmonertinib, including skin rash, leucopenia, thrombocytopenia, anemia, interstitial lung disease (ILD), and creatine phosphokinase (CPK) elevation. As can be seen from the (Table 2), furmonertinib exhibited better safety profile when compared with osimertinib and aumolertinib.

The FAVOUR study (NCT04858958) aimed to explore the real-world efficacy and safety of furmonertinib in advanced NSCLC patients harboring EGFR ex20ins. In the study, the most common TRAEs were diarrhea (26.4%) and rash (26.4%). Even in the group receiving 240 mg/d of furmonertinib, no TRAEs of grade ≥3 were found. Furthermore, there was no statistically significant difference observed in the incidence of TRAEs within the dosage range of 80–240 mg of furmonertinib (*p* = 0.271) (Sa et al., 2023). Thus, furmonertinib had a good safety profile without dose-dependent toxicity.

## Future development of furmonertinib

Osimertinib has been approved by U.S. FDA and China National Medical Products Administration (NMPA) as the adjuvant treatment in resected early-stage EGFR-classical mutated NSCLC based on the clinical study of ADAURA (Herbst et al., 2023). Several ongoing clinical trials are investigating the adjuvant treatment of furmonertinib for resectable EGFR-mutant NSCLC (Table 3). These trials include stage IA with high risk factors and stage IB (NCT05445310), IB-ⅡA (ATHEM, NCT05165355),Ⅱ-ⅢA (FORWARD, NCT04853342), Ⅱ-ⅢB (NCT05987826), and IIIA-IIIB (N1-N2) (FRONT, NCT04965831). Additionally, clinical trials are being initiated to explore the neoadjuvant role of furmonertinib in combination with bevacizumab (NCT 05503667) or cisplatin/pemetrexed (FORESEE, NCT05430802) for resectable and potentially resectable stage III-IVA or stage IIIA-IIIB EGFR mutant NSCLC in China.

**TABLE 3:** Ongoing clinical study of furmonertinib treatment for EGFR-mutant NSCLC.

Study	Phase	Title	Intervention/Treatment	Endpoint
NCT05445310	Phase 2	Adjuvant Furmonertinib in Stage IA With High Risk Factors and Stage IB Non-small Cell Lung Cancer: a Prospective Single-arm Study	Furmonertinib (80 mg/d) for 3 years	3-year DFS rate
NCT05165355 (ATHEM)	Phase 2	A Phase II, Single-armed Study to Assess the Efficacy and Safety of Furmonertinib in Patients With Epidermal Growth Factor Receptor Mutation Positive Stage IB-IIA Non-small Cell Lung Carcinoma, Following Complete Tumor Resection	Furmonertinib (80 mg/d) for 2 years	2-year DFS rate
NCT04853342 (FORWARD)	Phase 3	To Assess the Efficacy and Safety of Furmonertinib Versus Placebo, in Patients With Epidermal Growth Factor Receptor Mutation Positive Stage II-IIIA Non-small Cell Lung Carcinoma, Following Complete Tumor Resection With or Without Adjuvant Chemotherapy	Furmonertinib Vs. Placebo	Efficacy and Safety
NCT05987826	Phase 2	Furmonertinib Mesylate Neoadjuvant Treatment of Resectable Stage Ⅱ-ⅢB Non-small Cell Lung Cancer Patients With Epidermal Growth Factor Receptor (EGFR)Sensitive Mutation: a Prospective, Muliticenter, Open Label, Phase Ⅱ Single-arm Study	Furmonertinib (80 mg/d) for 8 weeks	Efficacy and Safety
NCT04965831 (FRONT)	Phase 2	Furmonertinib Mesylate as Perioperation Therapy in Stage IIIA-IIIB (N1-N2) Resectable, EGFR Sensitizing Mutation Positive Lung Adenocarcinoma Patients: A Phase II, Single-arm, Open-label Clinical Study (FRONT)	Furmonertinib (80 mg/d)	Efficacy and Safety
NCT05503667	Phase 2	Neoadjuvant Furmonertinib Plus Bevacizumab or Furmonertinib Monotherapy for Resectable and Potentially Resectable Stage III-IVA EGFR Mutation-Positive Lung Adenocarcinoma: A Randomized, Controlled, Open-label, Single-center Phase II Clinical Trial	Furmonertinib (80 mg/d) plus bevacizumab	Efficacy and Safety
NCT05430802 (FORESEE)	Phase 2	Furmonertinib Combined With Cisplatin/Pemetrexed as Neoadjuvant Therapy in EGFR Mutated Stage IIIA-IIIB Resectable Non-small Cell Lung Cancer (FORESEE): a Prospective, Open-label, Single-arm, Phase 2 Study	Furmonertinib (80 mg/d) plus Cisplatin/Pemetrexed	Efficacy and Safety
NCT05548348	Phase 2	A Single Arm, Multicenter Study of First-line Furmonertinib Treatment in Patients With Advanced Epidermal Growth Factor Receptor Uncommon Mutation Positive Non-small Cell Lung Cancer	Furmonertinib (160 mg/d)	Efficacy and Safety

Furthermore, first-line treatment of furmonertinib is being launched in advanced NSCLC patients with uncommon EGFR-mutations (NCT05548348) (Table 3).

## Concluding remarks of furmonertinib

**FIGURE 1:**
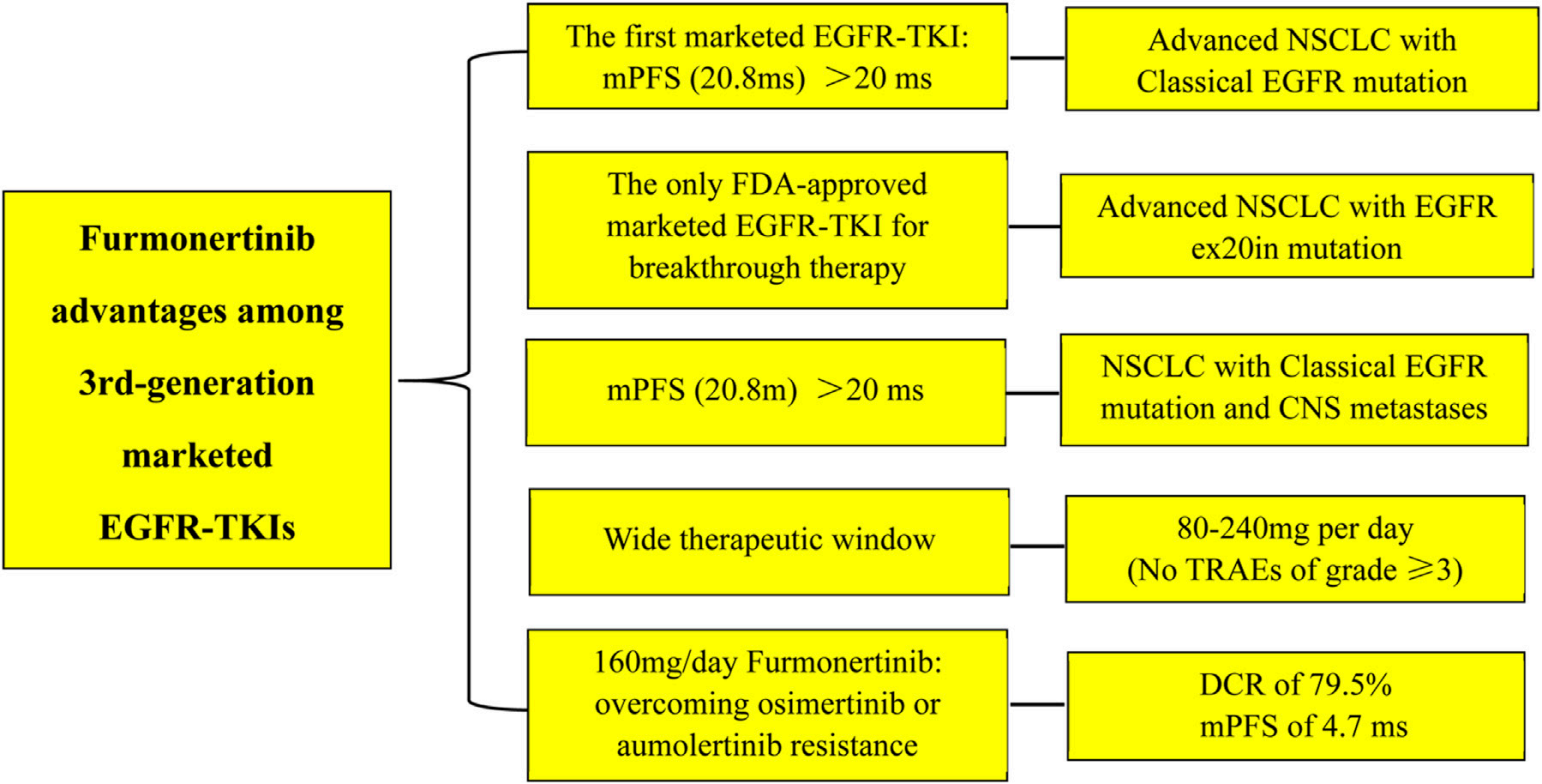
The advantages of furmonertinib among the 3rd-generations marketed EGFR-TKIs. Abbreviations: mPFS: median progression-free survival; ms, months; ex20in: exon 20 insertion; CNS: central nervous system; TRAEs: treatment-related adverse effects; DCR: disease control rate.

Furmonertinib, as a novel 3rd-generation EGFR-TKI, demonstrates excellent clinical efficacy, a good safety profile, and a wide therapeutic window (80mg–240 mg) in advanced NSCLC with EGFR classical and CNS metastases. Furthermore, furmonertinib shows superior efficacy in patients with exon 20 insertions mutations, which is unrivalled by osimertinib and aumolertinib. In particular, furmonertinib possesses better effect on NSCLC patients after osimertinib or aumolertinib resistance (Figure 1). There are grounds to believe that furmonertinib will be of promising prospect to treat the vast majority activating EGFR-mutant NSCLC.

## References

[B1] Chen W. Miao J. Wang Y. Xing W. Xu X. Wu R. (2023). Comparison of the efficacy and safety of first-line treatments for of advanced egfr mutation-positive non-small-cell lung cancer in asian populations: a systematic review and network meta-analysis. Front. Pharmacol. 14, 1212313. 10.3389/fphar.2023.1212313 37484016 PMC10358853

[B2] Cheng Y. He Y. Li W. Zhang H. L. Zhou Q. Wang B. (2021). Osimertinib versus comparator egfr tki as first-line treatment for egfr-mutated advanced nsclc: flaura China, a randomized study. Target Oncol. 16, 165–176. 10.1007/s11523-021-00794-6 33544337 PMC7935816

[B3] Chinese Association for Clinical Oncologists (2023). China clinical practice guideline for epidermal growth factor receptor tyrosine kinase inhibitors in stage ⅳ non-small cell lung cancer (version 2023). Zhonghua Yi Xue Za Zhi 103, 3160–3173. 10.3760/cma.j.cn112137-20230505-00725 37879869

[B4] Deng Y. Yang C. Liu W. Cai S. Guo X. (2022). Aumolertinib-based comprehensive treatment for an uncommon site of egfr exon 20 insertion mutations with multiple metastases non-small cell lung cancer: a case report. Anticancer Drugs 33, 406–412. 10.1097/CAD.0000000000001274 35266890

[B5] Elamin Y. Y. Robichaux J. P. Carter B. W. Altan M. Tran H. Gibbons D. L. (2022). Poziotinib for egfr exon 20-mutant nsclc: clinical efficacy, resistance mechanisms, and impact of insertion location on drug sensitivity. Cancer Cell 40, 754–767.e6. 10.1016/j.ccell.2022.06.006 35820397 PMC9667883

[B6] Herbst R. S. Wu Y. L. John T. Grohe C. Majem M. Wang J. (2023). Adjuvant osimertinib for resected egfr-mutated stage ib-iiia non-small-cell lung cancer: updated results from the phase iii randomized adaura trial. J. Clin. Oncol. 41, 1830–1840. 10.1200/JCO.22.02186 36720083 PMC10082285

[B7] Hou J. Li H. Ma S. He Z. Yang S. Hao L. (2022). Egfr exon 20 insertion mutations in advanced non-small-cell lung cancer: current status and perspectives. Biomark. Res. 10, 21. 10.1186/s40364-022-00372-6 35418149 PMC9008900

[B8] Hu X. Zhang S. Ma Z. Feng J. Wu L. Lv D. (2023). Central nervous system efficacy of furmonertinib (ast2818) in patients with egfr t790m mutated non-small cell lung cancer: a pooled analysis from two phase 2 studies. Bmc Med. 21, 164. 10.1186/s12916-023-02865-z 37118803 PMC10148399

[B9] Li W. Q. Cui J. W. (2020). Non-small cell lung cancer patients with ex19del or exon 21 l858r mutation: distinct mechanisms, different efficacies to treatments. J. Cancer Res. Clin. Oncol. 146, 2329–2338. 10.1007/s00432-020-03296-6 32596787 PMC11804701

[B10] Lu S. Dong X. Jian H. Chen J. Chen G. Sun Y. (2022). Aeneas: a randomized phase iii trial of aumolertinib versus gefitinib as first-line therapy for locally advanced or metastaticnon-small-cell lung cancer with egfr exon 19 deletion or l858r mutations. J. Clin. Oncol. 40, 3162–3171. 10.1200/JCO.21.02641 35580297 PMC9509093

[B11] Meng J. Zhang H. Bao J. J. Chen Z. D. Liu X. Y. Zhang Y. F. (2022). Metabolic disposition of the egfr covalent inhibitor furmonertinib in humans. Acta Pharmacol. Sin. 43, 494–503. 10.1038/s41401-021-00667-8 33927359 PMC8791928

[B12] Musib L. Kowanetz M. Li Q. (2022). Furmonertinib is an oral, irreversible, highly brain-penetrant pan-egfr mutant inhibitor with activity against classical and atypical egfr mutations. Chicago, Illinois, USA: North American Conference in Lung Cancer, 23–25.

[B13] Park K. Haura E. B. Leighl N. B. Mitchell P. Shu C. A. Girard N. (2021). Amivantamab in egfr exon 20 insertion-mutated non-small-cell lung cancer progressing on platinum chemotherapy: initial results from the chrysalis phase i study. J. Clin. Oncol. 39, 3391–3402. 10.1200/JCO.21.00662 34339292 PMC8791812

[B14] Qi R. Fu X. Yu Y. Xu H. Shen M. He S. (2023). Efficacy and safety of re-challenging 160 mg furmonertinib for advanced nsclc after resistance to third-generation egfr-tkis targeted agents: a real-world study. Lung Cancer 184, 107346. 10.1016/j.lungcan.2023.107346 37604026

[B15] Ramalingam S. S. Vansteenkiste J. Planchard D. Cho B. C. Gray J. E. Ohe Y. (2020). Overall survival with osimertinib in untreated, egfr-mutated advanced nsclc. N. Engl. J. Med. 382, 41–50. 10.1056/NEJMoa1913662 31751012

[B16] Sa H. Shi Y. Ding C. Ma K. (2023). A real-world study of the efficacy and safety of furmonertinib for patients with non-small cell lung cancer with egfr exon 20 insertion mutations. J. Cancer Res. Clin. Oncol. 149, 7729–7742. 10.1007/s00432-023-04726-x 37004599 PMC11796691

[B17] Shi Y. Chen G. Wang X. Liu Y. Wu L. Hao Y. (2022a). Central nervous system efficacy of furmonertinib (ast2818) versus gefitinib as first-line treatment for egfr-mutated nsclc: results from the furlong study. J. Thorac. Oncol. 17, 1297–1305. 10.1016/j.jtho.2022.07.1143 35932953

[B18] Shi Y. Chen G. Wang X. Liu Y. Wu L. Hao Y. (2022b). Furmonertinib (ast2818) versus gefitinib as first-line therapy for Chinese patients with locally advanced or metastatic egfr mutation-positive non-small-cell lung cancer (furlong): a multicentre, double-blind, randomised phase 3 study. Lancet Respir. Med. 10, 1019–1028. 10.1016/S2213-2600(22)00168-0 35662408

[B19] Shi Y. Zhang S. Hu X. Feng J. Ma Z. Zhou J. (2020). Safety, clinical activity, and pharmacokinetics of alflutinib (ast2818) in patients with advanced nsclc with egfr t790m mutation. J. Thorac. Oncol. 15, 1015–1026. 10.1016/j.jtho.2020.01.010 32007598

[B20] Soria J. C. Ohe Y. Vansteenkiste J. Reungwetwattana T. Chewaskulyong B. Lee K. H. (2018). Osimertinib in untreated egfr-mutated advanced non-small-cell lung cancer. N. Engl. J. Med. 378, 113–125. 10.1056/NEJMoa1713137 29151359

[B21] van Veggel B. Madeira R. S. J. Hashemi S. Paats M. S. Monkhorst K. Heideman D. (2020). Osimertinib treatment for patients with egfr exon 20 mutation positive non-small cell lung cancer. Lung Cancer 141, 9–13. 10.1016/j.lungcan.2019.12.013 31926441

[B22] Villaruz L. C. Wang X. Bertino E. M. Gu L. Antonia S. J. Burns T. F. (2023). A single-arm, multicenter, phase ii trial of osimertinib in patients with epidermal growth factor receptor exon 18 g719x, exon 20 s768i, or exon 21 l861q mutations. Esmo Open 8, 101183. 10.1016/j.esmoop.2023.101183 36905787 PMC10163152

[B23] Wu Y. L. Zhou Q. (2023). Combination therapy for egfr-mutated lung cancer. N. Engl. J. Med. 389, 2005–2007. 10.1056/NEJMe2311559 37937797

[B24] Yang G. J. Li J. Xu H. Y. Sun Y. Liu L. Li H. S. (2021). Osimertinib for Chinese advanced non-small cell lung cancer patients harboring diverse egfr exon 20 insertion mutations. Lung Cancer 152, 39–48. 10.1016/j.lungcan.2020.11.027 33341538

[B25] Yang J. C. Sequist L. V. Geater S. L. Tsai C. M. Mok T. S. Schuler M. (2015). Clinical activity of afatinib in patients with advanced non-small-cell lung cancer harbouring uncommon egfr mutations: a combined post-hoc analysis of lux-lung 2, lux-lung 3, and lux-lung 6. Lancet Oncol. 16, 830–838. 10.1016/S1470-2045(15)00026-1 26051236

[B26] Yasuda H. Ichihara E. Sakakibara-Konishi J. Zenke Y. Takeuchi S. Morise M. (2021). A phase i/ii study of osimertinib in egfr exon 20 insertion mutation-positive non-small cell lung cancer. Lung Cancer 162, 140–146. 10.1016/j.lungcan.2021.10.006 34808485

[B27] Zhao Y. Su C. Shi L. Luo W. Liu Z. Liang C. (2023). Case report: the effective treatment of patients in advanced no-small cell lung cancer patients with egfr g719x/s768i/l861q and acquired met amplification: a case series and literature review. Front. Oncol. 13, 1126325. 10.3389/fonc.2023.1126325 36910616 PMC9992534

[B28] Zhou C. Ramalingam S. S. Kim T. M. Kim S. W. Yang J. C. Riely G. J. (2021). Treatment outcomes and safety of mobocertinib in platinum-pretreated patients with egfr exon 20 insertion-positive metastatic non-small cell lung cancer: a phase 1/2 open-label nonrandomized clinical trial. Jama Oncol. 7, e214761. 10.1001/jamaoncol.2021.4761 34647988 PMC8517885

